# The impact of the COVID‐19 pandemic on aquaculture research

**DOI:** 10.1111/jwas.12775

**Published:** 2021-02-15

**Authors:** Matt Slater

**Affiliations:** ^1^ Journal of the World Aquaculture Society

1

Few of us would have considered the possibility of a global pandemic at the beginning of 2020, and most of us had quite specific plans of what we wished to achieve in the year ahead. Yet as the year progressed, researchers in aquaculture in every corner of the globe faced increasing restrictions affecting their working lives.

The COVID‐19 pandemic has had tragic consequences including death, severe illness and extreme isolation in particular for many of our older and respected colleagues. It has thus caused a significant loss of knowledge and (so often underestimated) wisdom in aquaculture science. We must acknowledge this professional loss, and the personal loss of so many individuals around the world, before we begin to consider the impact on our working lives. Perhaps this will make us more circumspect about our own challenges, which while not trivial, are lesser than those of people suffering from COVID‐19 or caring for them.

During the year, JWAS was fortunate to publish an excellent editorial on recent studies of the commercial impacts of COVID‐19 on aquaculture, aquaponics, and allied businesses in the United States (van Senten et al. 2020). There is a dearth of such analysis for impacts on active researchers, despite the fact that these have been manifold. First, the pandemic restrictions have meant colleagues have been unable to access their research sites and laboratories for most, if not all, of the past year. This has negatively affected experimental setup and completion, data collection and analyses in many laboratories worldwide. The advancement of projects funded locally, nationally or at international levels has thus been seriously impacted over the past year.

The inability to travel has resulted in many colleagues also being unable to carry out activities within their international collaborations or to complete any tasks in the foreign nations where they had planned to work. Other projects have been unable to fill positions awarded to overseas postdocs or international scientists with severe effects on task completion. For those colleagues who were able to focus more on local and national projects in lieu of international travel, there may have been some compensation of the lost progress. However, observations suggest that this compensation has been negligible. How long this will continue and what it will mean in terms of scientific outputs in 2021 is hard to predict. Nonetheless, it is fair to say for many projects and researchers that 2020 was a lost year.

International travel has not only been impacted in terms of scientific exchange or work in other nations by researchers. Contributors and organisers are sorely aware that all international conferences for aquaculture have been cancelled or moved to online formats in the past year. This trend also looks very likely to continue for at least the first half of 2021. The impact of this is of course felt keenly by the editorial team at JWAS, with limitations to our ability to take in the excellent work being conducted by colleagues around the world. We are also unable to share our thoughts or garner your insights into current and future hot topics for aquaculture research. Not only have we lost the chance to network in person, there is a large impact on the entire scientific community in the lack of informal face‐to‐face exchanges in and around conference sessions all around the world. The implications for this are significant in terms of reduced information exchange within the aquaculture community worldwide. How far this has been compensated by online collaboration, online conferences and other digital forms of communication is hard to estimate. I certainly have greatly missed the formal and informal exchanges which I so value from national and international conferences, workshops, meetings and exchanges. Many of us unable to travel to our institutes, research sites and laboratories have experienced this loss of spontaneous opportunities to create and develop new ideas at a local level as well.

Many researchers are also responsible for the well‐being and advancement of early postdocs and students completing degrees alongside teaching courses at universities. The pandemic's impact on the student experience and learning, through rapidly implemented online courses, lacking the hands‐on lab sessions or practicals, and absence of on‐farm or on‐site experience is already large. It is extremely important in the coming year that we as researchers and mentors improve the student experience as much as we can. If possible, we must attempt to make up for the face‐to‐face time those students have been unable to enjoy over the past year. Ensuring the optimal education of our young and upcoming scientists and producers is an extremely important part of our response as academics to ensure that no long‐term negative impact of the pandemic is felt within our young scientific community.

When the pandemic began, I mused whether there would be a flood of manuscripts submitted to the journal from academics who were forced to spend time at home, unable to commence exciting new research or new experiments. Instead, they would be forced to finally analyze and write up data sets, some of which may have been neglected for some time. This has certainly been the case for me and some colleagues in the first months of the pandemic and may have led to an increase in submissions in the second and third quarter of the year for JWAS. This however is unlikely to continue, as exciting datasets run out and the motivation of those banished to endless home‐office time fades. The researchers in the aquaculture science community are increasingly desperate instead for some return to normality as soon as possible.

Exploring what we can, or were forced to, do differently during the pandemic is important. For many, it has been surprising how much can be achieved in online meetings and conferences especially when developing new content or summarizing important subject areas. Existing networks and existing collaborations can also be kept active and vibrant at a distance using the variety of tools available for digital information exchange. Yet development of new networks and working relationships via digital or online platforms has proven much more difficult. Where physical research has been limited by lab, site or country access, some modeling and artificial intelligence approaches have shown real promise. These positives should be built on, if and when the restrictions are lifted. Many other positive insights and efficiencies have come with the pandemic. It is important that we acknowledge them and learn from them to improve our outputs and our services to those we work with and those we teach.

It remains very difficult to offer advice or ideas about the year ahead. Nonetheless, it is important to note that adversity often creates opportunities for rapid change, positive developments, new ideas and innovation. We must apply the diverse skills of our scientific community to ensure that the damage done to aquaculture is not long‐term and that the lessons learned in terms of vulnerability of supply chains and aquaculture production, along with aquaculture's importance in times of adversity are not lost. Young researchers, especially doctorate and early postdocs must “catch the curve” this year. They should not be negatively influenced in their career development or choices after a year of pandemic restrictions. Equally, we must ensure the continued growth of active scientific research and quality literature around aquaculture, which we have been so lucky to be involved in over the past decades.

So as the year commences with exciting opportunities and interesting times ahead, let us all take stock, learn and hope for a 2021 filled with positive new beginnings.

Matt Slater

Executive Editor

Journal of the World Aquaculture Society
